# Preoperative mental health predicts early satisfaction but not two-year knee-specific functional improvement after unicompartmental knee arthroplasty: an analysis of a robotic-assisted cohort

**DOI:** 10.1186/s42836-026-00448-w

**Published:** 2026-11-11

**Authors:** Yong Ng, Jeremy Tze En Lim, Isaac Chan, Elliot Jun Wei Goh, Darren Keng Jin Tay, Hee Nee Pang, Seng-Jin Yeo, Ming Han Lincoln Liow

**Affiliations:** 1 Department of Orthopaedics, Singapore General Hospital, Singapore Health Services, Singapore, Singapore; 2 Duke-NUS Medical School, Singapore, Singapore; 3 Lee Kong Chian School of Medicine, Nanyang Technological University, Singapore, Singapore

**Keywords:** Unicompartmental Knee Arthroplasty, Robotic-Assisted, Mental Health, Satisfaction, Patient-Reported Outcomes

## Abstract

**Background:**

Preoperative mental health is an important determinant of patient-reported outcome measures (PROMs), but its influence in unicompartmental knee arthroplasty (UKA) remains poorly characterized. In this robotic-assisted UKA cohort, we determined the effect of poor preoperative mental health on PROMs at six months and two years, described the change in mental health after surgery, and whether preoperative mental health predicted satisfaction and expectation fulfilment.

**Methods:**

Prospectively collected registry data on 241 consecutive robotic-assisted medial UKAs performed between 2017 and 2024 were reviewed. Of these, 121 formed the primary complete-case cohort, and patients were stratified by preoperative 36-item Short Form Health Survey (SF-36) Mental Component Summary (MCS) into poor mental health (MCS < 50, *n* = 51) and good mental health (MCS ≥ 50, *n* = 70). Outcomes included the Knee Society Function and Knee Score (KSFS & KSKS) and Oxford Knee Score (OKS), SF-36 Physical Component Summary (PCS), satisfaction, and expectation fulfillment. Multivariable logistic regression identified predictors of satisfaction, expectation fulfilment, and mental health recovery.

**Results:**

Mean preoperative MCS was 41.2 in the low-MCS group and 58.2 in the high-MCS group (*p* < 0.001), with the former achieving greater MCS improvement at six months and two years (*p* < 0.05). Despite a lower preoperative KSKS and a higher (worse) OKS (*p* < 0.05) in the low-MCS group, both groups demonstrated comparable improvements in KSFS, KSKS, and OKS. However, divergent global physical recovery was observed at two years, with the high-MCS group achieving greater improvements in the SF-36 PCS compared to the low-MCS group (Δ 17.0 versus 11.1, *p* = 0.006; MCID attainment 85.7% versus 66.7%, *p* = 0.024; ANCOVA-adjusted *p* = 0.002). At six months, satisfaction (82.4 versus 97.1%; *p* = 0.008) and expectation fulfillment (70.6 versus 90.0%; *p* = 0.008) were significantly lower in the low-MCS group, while no difference was detected at two years. In exploratory subgroup analysis, persistently low MCS patients had significantly worse two-year PROMs than the high-MCS group. Preoperative MCS independently predicted six-month satisfaction (OR 1.078; *p* = 0.005) and expectation fulfillment (OR 1.061; *p* = 0.005). Among low-MCS patients, six-month MCS was associated with two-year mental health normalization (OR 1.083; *p* = 0.008), with an internally derived cutoff of MCS ≥ 53 (area under the curve [AUC], 0.783).

**Conclusions:**

Within this robotic-assisted UKA cohort, patients with poor preoperative mental health reported lower early satisfaction, but still achieved meaningful mental health recovery and knee-specific functional gains comparable to those with better preoperative mental health by two years, with no detectable difference in satisfaction at two years. Poor preoperative mental health should not preclude appropriately selected patients from undergoing UKA. The six-month MCS is an internally derived exploratory signal that may help identify patients warranting closer monitoring.

**Supplementary Information:**

The online version contains supplementary material available at https://doi.org/10.1186/s42836-026-00448-w.

## Introduction

Unicompartmental knee arthroplasty (UKA) is a well-established surgical option for unicompartmental knee osteoarthritis, offering advantages over total knee arthroplasty (TKA) in terms of preserved bone stock, faster rehabilitation, and superior patient-reported satisfaction in appropriately selected patients [1, 2].

Robotic-assisted UKA systems allow precise intraoperative planning, bone resection, and implant positioning, and have been associated with improved component alignment and fewer alignment outliers than conventional technique [3–7]. Independent of surgical technique, patient-related factors account for much of the residual variation in outcome after arthroplasty. Among these factors, preoperative mental health has emerged as one of the most consistently reported predictors of outcome. In the total knee and hip arthroplasty literature, several articles have examined this relationship and found that poorer preoperative mental health was associated with lower postoperative scores, diminished functional gains, and greater dissatisfaction [8–11]. This relationship is likely multifactorial, reflecting differences in pain-related fear, central sensitization, illness beliefs, and expectation-outcome discordance.

While most studies have shown that preoperative mental health has been associated with worse outcomes after TKA, evidence on psychosocial predictors in UKA remains relatively sparse [12–15]. Given the differences in surgical characteristics, postoperative outcomes, and patient demographics between TKA and UKA, it cannot be assumed that findings from the TKA literature apply directly to UKA [2, 16–20]. Goh et al. found that conventional UKA patients with preoperative psychological distress demonstrated inferior ten-year outcomes, including lower Oxford Knee Scores (OKS) and reduced functional activity levels [14]. That study, however, was conducted before robotic-assisted techniques became widely adopted. Robotic assistance is now widely used and may itself shape how patients anticipate recovery [21], as patients often associate robotic arthroplasty with more accurate implant positioning and faster functional recovery [22]. It therefore remains unclear whether preoperative mental health predicts outcomes in contemporary UKA practice, and clarifying this relationship is clinically relevant for preoperative counselling and expectation management.

The primary aim of this study was to determine the effect of poor preoperative mental health on outcomes at six months and two years after UKA. Secondary aims were to describe the change in mental health after surgery and to determine whether preoperative mental health independently predicted patient satisfaction and expectation fulfilment. We hypothesized that preoperative mental health would be an important determinant of postoperative clinical outcomes and satisfaction.

## Methods

### Study design

**Fig. 1:**
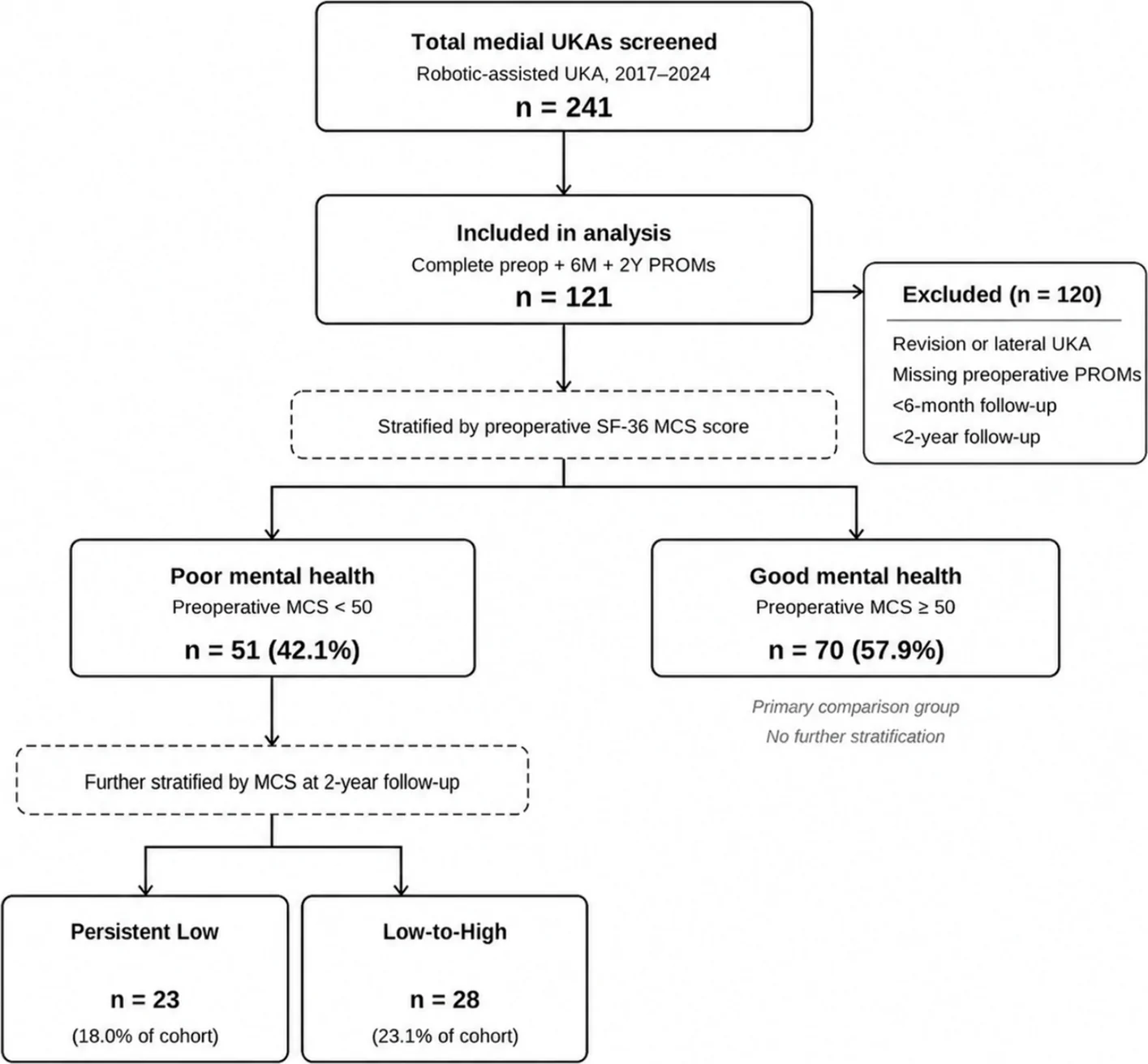
Patient inclusion flowchart and stratification by preoperative mental health status. MCS, mental component summary; PROMs, patient-reported outcome measures; SF-36, 36-item Short-Form health survey; UKA, unicompartmental knee arthroplasty

This was a retrospective, single-center analysis of prospectively collected registry data from a tertiary referral institution. Two hundred and forty-one consecutive medial UKAs performed from 2017 to 2024 met the following criteria: (1) primary robotic-assisted medial UKA; (2) diagnosis of isolated medial compartment knee osteoarthritis; and (3) complete preoperative patient-reported outcome measures (PROMs), including the preoperative mental health score. Patients undergoing revision surgery, lateral UKA, or those with incomplete preoperative mental health data were excluded. Of these 241 UKAs, 121 also had complete PROM data at both six months and two years and constituted the primary complete-case cohort for all analyses; the remaining 120 had incomplete follow-up data (Fig. 1). Institutional Review Board approval (IRB No. 2024/4046) was granted for this retrospective study and carried out in accordance with the ethical standards laid down in the 1964 Declaration of Helsinki.

Preoperative demographic variables, including age, sex, and body mass index (BMI), were collected by trained personnel at the institution’s Orthopaedic Diagnostic Centre. Patients were evaluated at three time points: preoperatively, six months, and two years. Outcomes included the 36-item Short Form Health Survey Questionnaire (SF-36), Knee Society Knee Score (KSKS), Knee Society Function Score (KSFS), OKS, and range of motion (ROM). The eight SF-36 domains were summarized into two higher-order component scores: the Physical Component Score (PCS) and Mental Component Score (MCS) [23]. The MCS is a generic measure of mental health and psychological distress. It has been used as an acceptable screening measure, but it is not a diagnostic instrument for depression or any other psychiatric disorder, and is interpreted here as a measure of general mental health rather than of depression [24–26].

Based on preoperative MCS, the cohort was stratified into two groups: low-MCS (< 50), representing below-average mental health, and high-MCS (≥ 50), representing above-average mental health. This threshold has previously been used to identify individuals in the general population at risk of psychological distress [27–31]. An exploratory subgroup analysis was conducted within the low-MCS cohort, where patients were classified into two trajectory groups based on their MCS score at two-year follow-up: those who remained persistently distressed (Persistent Low; MCS < 50 at both baseline and two years; *n* = 23) and those who achieved mental health normalization (Low-to-High; MCS < 50 at baseline recovering to MCS ≥ 50 at two years; *n* = 28).

Established minimal clinically important difference (MCID) thresholds were applied to the major PROMs, and the proportions of patients meeting MCID were compared to help contextualize clinical significance. MCID was defined as one-half of the standard deviation of the change between the preoperative and postoperative scores for the study cohort, consistent with an accepted distribution-based approach [32]. This yielded thresholds at six months of 10.6 (KSKS), 10.6 (KSFS), 4.3 (OKS), 5.2 (SF-36 PCS) and 5.2 (SF-36 MCS) points, and at two years of 12.2 (KSKS), 10.4 (KSFS), 4.4 (OKS), 6.0 (SF-36 PCS) and 5.8 (SF-36 MCS) points. Patient satisfaction and expectation fulfillment were assessed using a method similar to that described by Bourne et al. [33]. Satisfaction was rated on a 6-point scale and dichotomized as dissatisfied (“terrible,” “poor,” or “fair”) or satisfied (“good,” “very good,” or “excellent”). Expectation fulfillment was rated on a 7-point scale and similarly dichotomized as fulfilled (“Yes, totally,” “Yes, almost totally,” or “Yes, quite a bit”) or unfulfilled (“More or less,” “No, not quite,” “No, far from it,” or “No, not at all”).

### Patient demographics

As shown in Table 1, the cohort was predominantly female and Chinese. Patients in the low-MCS group (*n* = 51) had worse baseline clinical measures, including KSKS, OKS (in which a higher score denotes a worse outcome), and ROM, than those in the high-MCS group (*n* = 70). Mean preoperative MCS was 41.2 ± 9.6 in the low-MCS group and 58.2 ± 4.8 in the high-MCS group (*p* < 0.001).

**Table 1:** Preoperative characteristics

Characteristic	MCS < 50 (*n* = 51)	MCS ≥ 50 (*n* = 70)	*p*-value
Age/years	61.5 ± 8.3	65.6 ± 8.4	**0.007**
BMI/kg m^−2^	27.3 ± 5.1	26.6 ± 4.1	0.445
**Sex**			
Women	39 (76.5)	51 (72.9)	0.680
Men	12 (23.5)	19 (27.1)	
**Race**			
Chinese	42 (82.4)	55 (78.6)	0.181
Malay	8 (15.7)	8 (11.4)	
Indian	0 (0.0)	6 (8.6)	
Others	1 (2.0)	1 (1.4)	
**Preoperative outcomes**			
Range of motion/°	120.0 ± 16.0	127.9 ± 11.7	**0.010**
KSKS	37.8 ± 13.2	44.5 ± 16.9	**0.025**
KSFS	55.7 ± 19.7	61.7 ± 17.9	0.050
OKS	35.5 ± 8.4	31.3 ± 7.5	**0.008**
SF-36 PCS	36.2 ± 9.2	34.5 ± 9.3	0.420
SF-36 MCS	41.2 ± 9.6	58.2 ± 4.8	**< 0.001**

Data presented as mean ± standard deviation or *n* (%). Bold *p*-values indicate statistical significance (*p* < 0.05). OKS—lower scores indicate better outcomes

*BMI* body mass index, *KSKS* Knee Society Knee Score, *KSFS* Knee Society Function Score, *OKS* Oxford Knee Score, *MCS* mental component summary, *PCS* physical component summary, *SF-36* 36-item Short-Form health survey

### Surgical technique

All procedures were performed by three fellowship-trained orthopaedic surgeons using the Mako robotic-arm assisted system and cemented fixed-bearing Restoris MCK medial unicompartmental implants (Stryker Orthopaedics, Mahwah, NJ, USA). Preoperative computed tomography was obtained to generate a patient-specific three-dimensional model, which was used to plan femoral and tibial component sizing, positioning, and bone preparation. Surgery was performed through a limited medial parapatellar approach without patellar subluxation under tourniquet control. After exposure and osteophyte clearance, femoral and tibial registration was performed, followed by intraoperative assessment of limb alignment and medial compartment gaps through flexion and extension stress poses. The preoperative plan was adjusted intraoperatively to achieve balanced flexion–extension gaps while avoiding overcorrection, with attention to matching the femoral component/anterior flange to the retained cartilage surface given the inlay design. Robotic-arm assisted bone preparation was then performed within the planned haptic boundaries, followed by trial reduction to confirm implant fit, tracking, range of motion, and gap balance. Final components were cemented, the wound was closed in layers, and all patients underwent a standardized postoperative rehabilitation protocol.

### Data analyses

Normality and homogeneity of variance were assessed using the Shapiro–Wilk and Levene’s tests, respectively. Continuous variables were compared using the Student’s t-test or Wilcoxon rank-sum test, as appropriate, and categorical variables were compared using the Chi-square test or Fisher’s exact test. Analysis of Covariance (ANCOVA) was performed, adjusting for covariates such as baseline outcome scores, age, sex, and BMI. Multivariable logistic regression was performed to examine the association of MCS with patient satisfaction and expectation fulfillment after adjusting for the same covariates. To evaluate the robustness of the primary findings, the same analyses were repeated in the expanded cohort of all 241 UKAs on an available-case basis, in which the number analysed at each timepoint varied according to data availability (Supplementary Tables S1–S3).

Within the low-MCS subgroup, univariable logistic regression was performed to identify preoperative and early postoperative factors associated with MCS recovery by two years. Receiver operating characteristic (ROC) curve analyses were performed, and the optimal threshold for each factor was determined using the Youden method, in which the cut-off point on the ROC curve corresponds to the highest combination of sensitivity and specificity. These analyses were exploratory, were derived and evaluated within the same small subgroup without external validation, and are reported as hypothesis-generating.

Although multiple PROMs were compared across three timepoints and within subgroups, no formal adjustment for multiple comparisons was applied to the primary between-group comparisons, but post-hoc pairwise comparisons across trajectory subgroups were Bonferroni-corrected. A concise post hoc power assessment was retained solely to caveat the two-year comparisons: based on observed proportions, the logistic regression analyses of satisfaction and expectation fulfillment had adequate power (> 80%) at six months but insufficient power (< 80%) at two years. Statistical significance was defined as *p* < 0.05. All statistical analyses were performed using Python (version 3.12; Python Software Foundation, Beaverton, OR, USA).

## Results

### Change in mental health after surgery

**Fig. 2:**
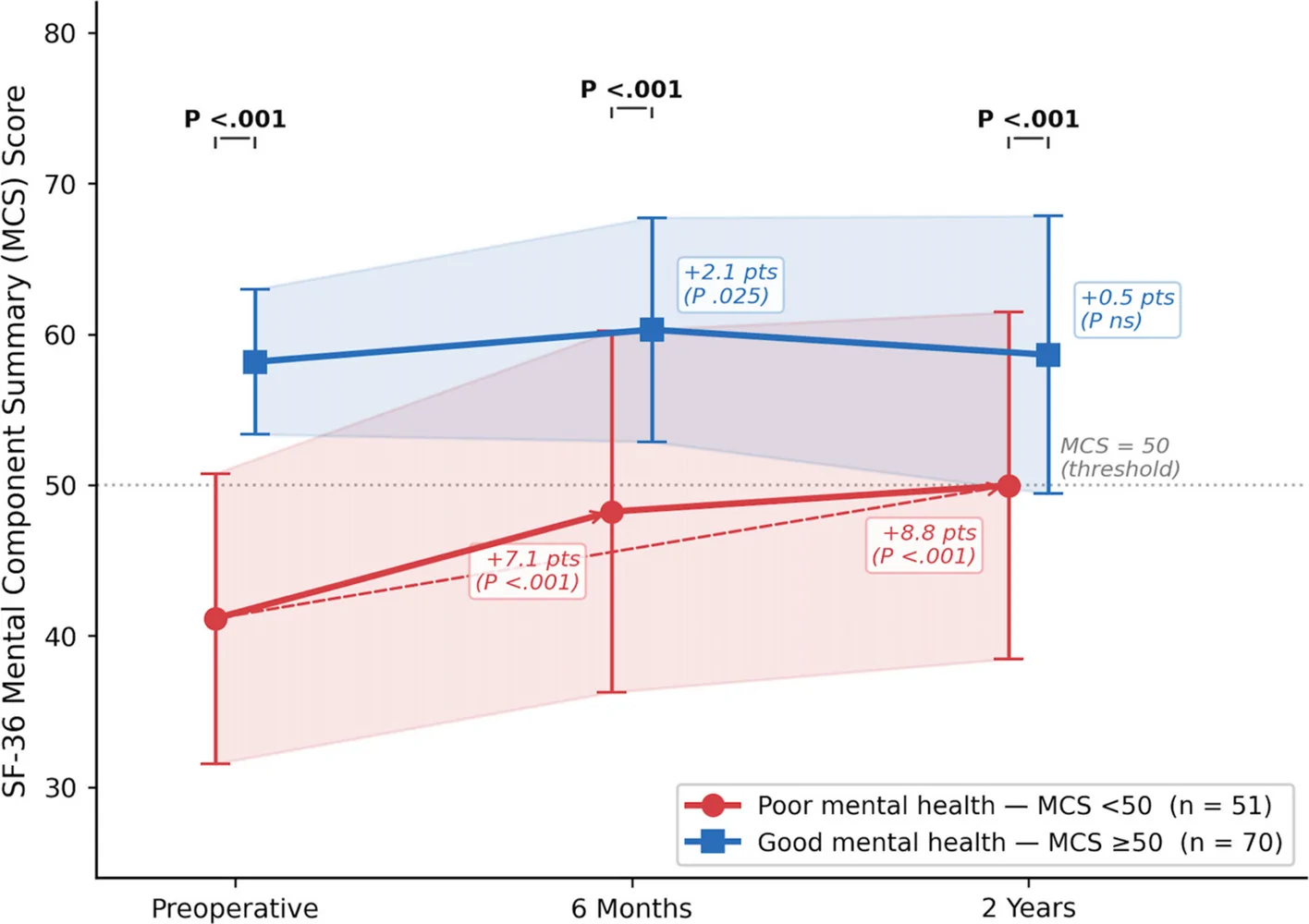
Longitudinal trajectory of the SF-36 Mental Component Summary score stratified by preoperative mental health status. Mean SF-36 MCS scores ± standard deviation are plotted at three time points: preoperative, 6 months, and 2 years postoperatively. Red circles represent patients with poor preoperative mental health and blue squares represent patients with good preoperative mental health. Shaded bands denote ± 1 standard deviation. Brackets indicate between-group significance at each time point. Delta annotations denote within-group change from baseline with corresponding *p*-values. The dotted horizontal line at MCS = 50 denotes the stratification threshold. MCS, mental component summary; SD, standard deviation; SF-36, 36-item Short-Form health survey

In the low-MCS group, patients’ mean MCS improved significantly by 7.1 ± 12.5 points at six months (*p* < 0.001) and by 8.8 ± 12.7 points at two years (*p* < 0.001). In contrast, within the high-MCS group, patients improved by 2.1 ± 7.8 points at six months (*p* = 0.025), with no significant change at two years (*p* = 0.296). Despite differential improvement in MCS, the high-MCS group maintained significantly higher MCS at all time points: at six months (60.3 ± 7.4 versus 48.2 ± 12.0, *p* < 0.001), and at two years (58.6 ± 9.2 versus 50.0 ± 11.5, *p* < 0.001) (Fig. 2).

### Effect of preoperative mental health on outcomes

Patients in the low-MCS group had worse baseline outcome scores than those in the high-MCS group. Despite these differences in preoperative scores, both groups showed comparable improvements in KSFS, KSKS, and OKS over time (*p* > 0.05). However, improvement in PCS at two years was greater in the high-MCS group than in the low-MCS group (17.0 ± 11.8 versus 11.1 ± 11.6, *p* = 0.006). Furthermore, at two years, a greater proportion of patients in the high-MCS group achieved the MCID for KSKS (92.9 versus 78.4%, *p* = 0.041) and PCS (85.7 versus 66.7%, *p* = 0.024) (Table 2). After adjusting for covariates, the high-MCS group demonstrated superior outcomes compared to the low-MCS group in KSKS, OKS, and PCS at two years (*p* < 0.05; Table 3). With respect to subjective outcomes, the high-MCS group had a higher proportion of patients who reported satisfaction and fulfilled expectations at six months (*p* < 0.05).

**Table 2:** Comparison of clinical outcomes and MCID achievement between low- and high-MCS groups

Outcome	MCS < 50 (*n* = 51)	MCS ≥ 50 (*n* = 70)	*p*-value
**Range of motion/°**			
Preoperative	120.0 ± 16.0	127.9 ± 11.7	**0.010**
6 months	122.7 ± 13.9	129.0 ± 13.1	**0.017**
2 years	126.9 ± 14.0	131.4 ± 12.7	0.072
Δ Preoperative to 6 months	3.2 ± 15.7	1.1 ± 11.2	0.414
Δ Preoperative to 2 years	7.0 ± 18.5	3.5 ± 10.6	0.266
**KSKS**			
Preoperative	37.8 ± 13.2	44.5 ± 16.9	**0.025**
6 months	84.5 ± 19.0	87.9 ± 13.2	0.664
2 years	79.0 ± 23.2	89.5 ± 13.4	**0.021**
Δ Preoperative to 6 months	46.9 ± 22.1	43.4 ± 20.5	0.296
Δ Preoperative to 2 years	40.5 ± 25.9	45.0 ± 23.2	0.385
Attained MCID at 6 months	46 (90.2)	67 (95.7)	0.279
Attained MCID at 2 years	40 (78.4)	65 (92.9)	**0.041**
**KSFS**			
Preoperative	55.7 ± 19.7	61.7 ± 17.9	0.050
6 months	73.2 ± 17.1	78.6 ± 15.2	**0.035**
2 years	77.1 ± 17.4	82.7 ± 17.6	0.064
Δ Preoperative to 6 months	17.6 ± 22.3	16.9 ± 20.5	0.716
Δ Preoperative to 2 years	21.4 ± 24.0	21.0 ± 18.4	0.909
Attained MCID at 6 months	26 (51.0)	30 (42.9)	0.484
Attained MCID at 2 years	31 (60.8)	45 (64.3)	0.839
**OKS**			
Preoperative	35.5 ± 8.4	31.3 ± 7.5	**0.008**
6 months	20.0 ± 5.9	18.9 ± 5.0	0.398
2 years	20.3 ± 6.4	17.3 ± 4.9	**0.003**
Δ Preoperative to 6 months	−15.5 ± 8.8	−12.3 ± 8.3	0.126
Δ Preoperative to 2 years	−15.3 ± 9.6	−14.0 ± 8.4	0.441
Attained MCID at 6 months	48 (94.1)	59 (84.3)	0.149
Attained MCID at 2 years	45 (88.2)	61 (87.1)	1.000
**SF-36 PCS**			
Preoperative	36.2 ± 9.2	34.5 ± 9.3	0.420
6 months	47.9 ± 9.5	49.1 ± 6.8	0.969
2 years	47.3 ± 9.1	51.6 ± 8.0	**0.001**
Δ Preoperative to 6 months	11.7 ± 9.5	14.6 ± 11.1	0.094
Δ Preoperative to 2 years	11.1 ± 11.6	17.0 ± 11.8	**0.006**
Attained MCID at 6 months	36 (70.6)	55 (78.6)	0.429
Attained MCID at 2 years	34 (66.7)	60 (85.7)	**0.024**
**SF-36 MCS**			
Preoperative	41.2 ± 9.6	58.2 ± 4.8	**< 0.001**
6 months	48.2 ± 12.0	60.3 ± 7.4	**< 0.001**
2 years	50.0 ± 11.5	58.6 ± 9.2	**< 0.001**
Δ Preoperative to 6 months	7.1 ± 12.5	2.1 ± 7.8	**0.015**
Δ Preoperative to 2 years	8.8 ± 12.7	0.5 ± 9.3	**< 0.001**
Attained MCID at 6 months	30 (58.8)	26 (37.1)	**0.030**
Attained MCID at 2 years	29 (56.9)	17 (24.3)	**0.001**
**Patient satisfaction**			
Satisfied at 6 months	42 (82.4)	68 (97.1)	**0.008**
Satisfied at 2 years	47 (92.2)	69 (98.6)	0.161
**Patient expectation**			
Expectation fulfilled at 6 months	36 (70.6)	63 (90.0)	**0.008**
Expectation fulfilled at 2 years	44 (86.3)	66 (94.3)	0.199

Data presented as mean ± standard deviation or *n* (%). Bold *p*-values indicate statistical significance. Δ represents the change from preoperative to 6 months or 2 years. *P*-values corresponding to Δ rows represent between-group comparisons of the change over time. OKS—lower scores indicate better outcomes

*KSKS* Knee Society Knee Score, *KSFS* Knee Society Function Score, *OKS* Oxford Knee Score, *MCID* minimal clinically important difference, *MCS* mental component summary, *PCS* physical component summary, *SF-36* 36-item Short-Form health survey

**Table 3:** Comparison of clinical outcomes between low and high MCS groups (ANCOVA-adjusted)

Outcome	MCS < 50 (*n* = 51)	MCS ≥ 50 (*n* = 70)	*p*-value
**Range of motion/°**			
6 months	125.4 ± 1.7	127.4 ± 1.4	0.359
2 years	128.4 ± 1.8	130.4 ± 1.5	0.398
**KSKS**			
6 months	85.5 ± 2.3	87.4 ± 1.9	0.540
2 years	78.0 ± 2.7	89.7 ± 2.3	**0.002**
**KSFS**			
6 months	73.8 ± 2.3	78.2 ± 1.9	0.152
2 years	77.5 ± 2.4	82.4 ± 2.0	0.133
**OKS**			
6 months	19.4 ± 0.8	19.4 ± 0.7	0.987
2 years	20.0 ± 0.8	17.5 ± 0.7	**0.021**
**SF-36 PCS**			
6 months	48.0 ± 1.1	49.0 ± 0.9	0.481
2 years	46.8 ± 1.2	51.9 ± 1.0	**0.002**

ANCOVA-adjusted analyses presented as adjusted mean ± standard error (SE). Adjusted means and SE are estimated at the grand mean of all covariates (corresponding baseline score, age, sex, and body mass index). Bold *p-*values indicate statistical significance. OKS—lower scores indicate better outcomes

*ANCOVA* Analysis of Covariance, *KSKS* Knee Society Knee Score, *KSFS* Knee Society Function Score, *OKS* Oxford Knee Score, *MCS* mental component summary, *PCS* physical component summary, *SF-36* 36-item Short-Form health survey

In the exploratory subgroup analysis, patients in the persistent low-MCS group demonstrated significant improvements in KSKS, KSFS, OKS, and PCS scores at both six months and two years (*p* < 0.05; Table 4). Descriptively, the persistent low-MCS group scored significantly lower than the high-MCS group across all assessed PROMs at two years (*p* < 0.05; Tables 5, 6 and 7). On univariable logistic regression within the low-MCS subgroup, six-month MCS was the only variable associated with mental health normalization (achieving MCS ≥ 50 by two years) (odds ratio [OR] 1.083; 95% confidence interval [CI] 1.021–1.150; *p* = 0.008) (Table 8). Six-month MCS discriminated recovery with an area under the curve (AUC) of 0.783 at an internally derived optimal cutoff of MCS ≥ 53 (Table 9).

**Table 4:** Longitudinal clinical outcomes in the persistent low-MCS group (*n* = 23)

Outcome	Preoperative	6 Months	2 Years	*p*-value (Preop vs 6 M)	*p*-value (Preop vs 2Y)
Range of Motion/°	120.65 ± 14.51	122.91 ± 16.33	125.13 ± 16.74	0.548	0.354
KSKS	36.87 ± 11.29	80.35 ± 21.41	72.48 ± 24.03	**< 0.001**	**< 0.001**
KSFS	58.04 ± 16.63	68.70 ± 19.14	70.65 ± 20.02	**0.002**	**0.016**
OKS	35.91 ± 7.51	21.00 ± 6.40	22.57 ± 6.97	**< 0.001**	**< 0.001**
SF-36 PCS	36.32 ± 9.42	48.74 ± 10.23	45.68 ± 9.81	**< 0.001**	**0.001**
SF-36 MCS	38.93 ± 10.39	42.97 ± 10.45	39.37 ± 7.84	0.126	0.858

Data presented as mean ± SD or *n* (%). Bold *p-*values indicate statistical significance. OKS—lower scores indicate better outcomes

*KSKS* Knee Society Knee Score, *KSFS* Knee Society Function Score, *OKS* Oxford Knee Score, *MCS* mental component summary, *PCS* physical component summary, *SF-36* 36-item Short-Form health survey

**Table 5:** Comparison of continuous clinical outcomes across MCS trajectory subgroups

Outcome	Persistent Low (*n* = 23)	Low-to-High (*n* = 28)	High-MCS (*n* = 70)	*p-*value
**Preoperative**				
Range of Motion/°	120.65 ± 14.51	119.37 ± 17.49	127.94 ± 11.71	**0.033**
KSKS	36.87 ± 11.29	38.54 ± 14.81	44.47 ± 16.91	0.079
KSFS	58.04 ± 16.63	53.75 ± 21.93	61.71 ± 17.87	0.143
OKS	35.91 ± 7.51	35.18 ± 9.21	31.26 ± 7.47	**0.020**
SF-36 PCS	36.32 ± 9.42	36.10 ± 9.26	34.53 ± 9.27	0.721
SF-36 MCS	38.93 ± 10.39	42.97 ± 8.70	58.17 ± 4.81	**< 0.001**
**6 Months**				
Range of Motion/°	122.91 ± 16.33	122.54 ± 11.94	129.00 ± 13.14	**0.049**
KSKS	80.35 ± 21.41	87.82 ± 16.33	87.91 ± 13.17	0.419
KSFS	68.70 ± 19.14	76.96 ± 14.55	78.64 ± 15.18	**0.026**
OKS	21.00 ± 6.40	19.14 ± 5.52	18.91 ± 5.00	0.429
SF-36 PCS	48.74 ± 10.23	47.15 ± 8.89	49.12 ± 6.79	0.441
SF-36 MCS	42.97 ± 10.45	52.56 ± 11.55	60.30 ± 7.44	**< 0.001**
**2 Years**				
Range of Motion/°	125.13 ± 16.74	128.29 ± 11.29	131.44 ± 12.72	0.188
KSKS	72.48 ± 24.03	84.36 ± 21.41	89.49 ± 13.42	**0.006**
KSFS	70.65 ± 20.02	82.32 ± 12.94	82.71 ± 17.56	**0.015**
OKS	22.57 ± 6.97	18.36 ± 5.37	17.30 ± 4.93	**< 0.001**
SF-36 PCS	45.68 ± 9.81	48.58 ± 8.44	51.55 ± 7.95	**0.004**
SF-36 MCS	39.37 ± 7.84	58.68 ± 4.51	58.64 ± 9.21	**< 0.001**

Data presented as mean ± SD. Bold *p-*values indicate statistical significance. OKS—lower scores indicate better outcomes

*KSKS* Knee Society Knee Score, *KSFS* Knee Society Function Score, *OKS* Oxford Knee Score, *MCS* mental component summary, *PCS* physical component summary, *SF-36* 36-item Short-Form health survey

**Table 6:** MCID attainment and patient-reported satisfaction and expectation fulfilment across MCS trajectory subgroups

Outcome	Persistent Low (*n* = 23)	Low-to-High (*n* = 28)	High-MCS (*n* = 70)	*p*-value
**6 Months**				
Satisfied, n (%)	18 (78.3)	24 (85.7)	68 (97.1)	**0.013**
Expectation fulfilled, n (%)	16 (69.6)	20 (71.4)	63 (90.0)	**0.024**
MCID attained, KSKS	22 (95.7)	26 (92.9)	67 (95.7)	0.832
MCID attained, KSFS	10 (43.5)	16 (57.1)	30 (42.9)	0.421
MCID attained, OKS	22 (95.7)	26 (92.9)	59 (84.3)	0.237
MCID attained, SF-36 PCS	16 (69.6)	20 (71.4)	55 (78.6)	0.597
**2 Years**				
Satisfied, n (%)	20 (87.0)	27 (96.4)	69 (98.6)	0.052
Expectation fulfilled, n (%)	19 (82.6)	25 (89.3)	66 (94.3)	0.226
MCID attained, KSKS	18 (78.3)	24 (85.7)	65 (92.9)	0.145
MCID attained, KSFS	11 (47.8)	21 (75.0)	56 (80.0)	**0.010**
MCID attained, OKS	20 (87.0)	25 (89.3)	61 (87.1)	0.953
MCID attained, SF-36 PCS	15 (65.2)	19 (67.9)	60 (85.7)	**0.045**

Data presented as n (%). Bold *p*-values indicate statistical significance

*KSKS* Knee Society Knee Score, *KSFS* Knee Society Function Score, *OKS* Oxford Knee Score, *MCID* minimal clinically important difference, *MCS* mental component summary, *PCS* physical component summary, *SF-36* 36-item Short-Form health survey

**Table 7:** Post-Hoc pairwise comparisons for outcomes reaching significance (*p* < 0.05)

Outcome	Persistent Low versus Low-to-High (*p*-value)	Persistent Low versus High-MCS (*p*-value)	Low-to-High versus High-MCS (*p*-value)
**Preoperative**			
Range of Motion/°	1.000	0.208	0.066
OKS	1.000	**0.027**	0.276
SF-36 MCS	1.000	**< 0.001**	**< 0.001**
**6 Months**			
Range of Motion/°	1.000	0.400	0.070
KSFS	0.278	**0.021**	1.000
SF-36 MCS	**0.010**	**< 0.001**	**0.004**
Satisfaction (proportion satisfied)	1.000	**0.028**	0.162
Expectation fulfilment (proportion fulfilled)	1.000	0.114	0.091
**2 Years**			
KSKS	0.077	**0.004**	1.000
KSFS	0.077	**0.013**	1.000
OKS	**0.008**	**< 0.001**	1.000
SF-36 PCS	1.000	**0.013**	0.052
SF-36 MCS	**< 0.001**	**< 0.001**	1.000
MCID attainment, KSFS	0.239	**0.019**	1.000
MCID attainment, SF-36 PCS	1.000	0.191	0.158

Dunn test with Bonferroni correction applied for continuous outcomes. Fisher’s exact test with Bonferroni correction applied for MCID outcomes. Bold *p*-values indicate statistical significance

*KSKS*, Knee Society Knee Score; *KSFS*, Knee Society Function Score; *OKS*, Oxford Knee Score; *MCS*, mental component summary; *PCS*, physical component summary; *SF-36*, 36-item Short-Form health survey; *MCID*, minimal clinically important difference

**Table 8:** Univariable logistic regression: preoperative and early postoperative predictors of MCS recovery

Variable	OR	95% CI Lower	95% CI Upper	95% CI	*p*-value
SF-36 MCS (Preoperative)	1.047	0.984	1.114	0.984–1.114	0.144
SF-36 PCS (Preoperative)	0.997	0.939	1.059	0.939–1.059	0.931
OKS	0.989	0.926	1.057	0.926–1.057	0.754
KSKS	1.010	0.967	1.055	0.967–1.055	0.656
KSFS	0.989	0.960	1.018	0.960–1.018	0.436
Range of Motion/°	0.995	0.960	1.031	0.960–1.031	0.776
Age/years	0.990	0.926	1.059	0.926–1.059	0.779
BMI/kg m^−2^	1.039	0.930	1.160	0.930–1.160	0.498
Sex (Men)	0.497	0.134	1.847	0.134–1.847	0.297
SF-36 MCS (6 months)	1.083	1.021	1.150	1.021–1.150	**0.008**

Bold *p-*values indicate statistical significance

*OR* odds ratio, *CI* confidence interval, *BMI* body mass index, *OKS* Oxford Knee Score, *KSKS* Knee Society Knee Score, *KSFS* Knee Society Function Score, *MCS* mental component summary, *PCS* physical component summary, *SF-36* 36-item Short-Form health survey

**Table 9:** Receiver Operating Characteristic (ROC) Analysis for Prediction of Mental Health Recovery

Predictor	AUC	Optimal Threshold	Sensitivity (%)	Specificity (%)	PPV (%)	NPV (%)
SF-36 MCS (Preoperative)	0.640	MCS_0_ ≥ 46.1	57.1	73.9	-	-
SF-36 MCS (6 months)	0.783	MCS_6_ ≥ 53.0	71.4	91.3	90.9	72.4

ROC analysis was performed within patients with preoperative MCS < 50 (*n* = 51)

*PPV* positive predictive value, *NPV* negative predictive value, *AUC* area under the receiver operating characteristic curve, *MCS* mental component summary, *SF-36* 36-item Short-Form health survey

### Preoperative mental health as a predictor of satisfaction and expectation fulfillment

In multivariable analysis, preoperative MCS was the only independent predictor of achieving patient satisfaction (OR 1.078; *p* = 0.005) and expectation fulfillment (OR 1.061; *p* = 0.005) at six months. However, the influence of preoperative mental health on satisfaction and expectation fulfillment attenuated by two years (Tables 10 and 11).

**Table 10:** Multivariable logistic regression for achieving patient satisfaction

Variable	OR	95% CI		*p*-value
		**Lower**	**Upper**	
**At 6 months**				
Preoperative SF-36 MCS	1.078	1.024	1.135	**0.005**
Age	1.071	0.981	1.170	0.126
Sex (Women)	1.114	0.233	5.336	0.892
**Sex (Men)**				
BMI	1.058	0.912	1.227	0.455
**At 2 years**				
Preoperative SF-36 MCS	1.054	0.987	1.125	0.118
Age	1.059	0.937	1.197	0.357
Sex (Women)	0.537	0.077	3.736	0.530
**Sex (Men)**				
BMI	1.069	0.865	1.321	0.537

Bold *p-*values indicate statistical significance. Bold variable labels denote the reference category

*BMI* body mass index, *CI* confidence interval, *MCS* mental component summary, *OR* odds ratio, *SF-36* 36-item Short-Form health survey

**Table 11:** Multivariable logistic regression for achieving expectation fulfillment

Variable	OR	95% CI		*p*-value
		**Lower**	**Upper**	
**At 6 months**				
Preoperative SF-36 MCS	1.061	1.019	1.106	**0.005**
Age	1.036	0.975	1.102	0.254
Sex (Women)	1.362	0.418	4.437	0.609
**Sex (Men)**				
BMI	1.030	0.925	1.148	0.590
**At 2 years**				
Preoperative SF-36 MCS	1.040	0.990	1.092	0.117
Age	0.987	0.911	1.069	0.750
Sex (Women)	0.449	0.113	1.782	0.255
**Sex (Men)**				
BMI	1.152	0.978	1.358	0.090

Bold *p-*values indicate statistical significance. Bold variable labels denote the reference category

*BMI* body mass index, *CI* confidence interval, *MCS* mental component summary, *OR* odds ratio, *SF-36* 36-item Short-Form health survey

## Discussion

This study suggested that preoperative mental health was associated with both objective and subjective PROMs following UKA, supporting our hypothesis. Although patients with poorer preoperative mental health had worse postoperative PROM scores, the magnitude of improvement in knee-specific outcomes (KSFS, KSKS, and OKS) was comparable between groups. In contrast, improvement in global physical health (PCS) was greater in patients with higher preoperative MCS. Sensitivity analyses in the expanded cohort of all 241 UKAs broadly confirmed these findings, demonstrating comparable improvements in knee-specific outcomes between MCS groups and persistent associations between preoperative MCS and early satisfaction and expectation fulfilment.

Our findings were consistent with the broader total joint arthroplasty (TJA) literature, which has shown that preoperative psychological status is associated with postoperative PROMs and satisfaction [11, 12, 14, 34–36]. In a study of 1,473 patients, Goh et al. found that patients with poor mental health reported greater dissatisfaction after UKA despite experiencing greater improvement in knee function [12]. Studies using similar SF-36 MCS thresholds have likewise shown that psychological distress predicts worse PROMs after TJA [29, 37]. More recently, Sorel et al. reported in a systematic review that preoperative psychological distress appeared to adversely affect pain and function after TKA [31]. However, only one study included in that review had a clinical follow-up beyond one year [30], and no review to date has looked at the isolated effect of preoperative mental health on pain and function after UKA.

The present study showed that the proportion of patients with MCS < 50 decreased from 42.1% (51/121) preoperatively to 27.3% (33/121) at two-year follow-up. Patients with poorer preoperative mental health (MCS < 50) demonstrated a substantially greater MCS improvement (+ 8.8 versus + 0.5 points) compared to those with better preoperative mental health (MCS ≥ 50). Nevertheless, the higher preoperative mental health group continued to have greater MCS at all time points. Psychological distress in patients with advanced osteoarthritis often arises secondarily from loss of functional independence and the social isolation associated with severe mobility limitation [38]. Improvement in mental health after addressing the mechanical source of pain is consistent with the wider TJA literature [39, 40]. Because every patient in this cohort was treated with robotic assistance and no conventional comparator group was available, these observations describe recovery within a robotic-assisted cohort and cannot be attributed to the robotic platform itself.

The exploratory subgroup analysis suggests that low preoperative MCS may encompass two distinct trajectories: patients whose mental health normalizes after surgery and those with persistent low MCS despite improvement in knee-specific outcomes. This distinction may be clinically relevant, as statistically significant improvement does not necessarily equate to attainment of a patient-acceptable symptom state (PASS). Using the PASS thresholds defined by Goh et al. after UKA [41], patients in the persistent low-MCS group improved from baseline but appeared to remain below clinically acceptable benchmarks for several PROMs at two years. These subgroup findings are hypothesis-generating and do not establish a treatment pathway.

While baseline demographic characteristics and preoperative PROMs did not clearly differentiate these groups, the six-month postoperative visit may provide a practical opportunity for further risk stratification. In this cohort, patients whose six-month MCS had reached ≥ 53 were more likely to have normalized by two years. This threshold was derived internally within a subgroup of 51 patients. Given that the predictor and the outcome are the same instrument measured at two time points, this discrimination partly reflects the correlation of a measure with itself rather than independent prediction. Pending external validation, a lower six-month MCS may reasonably prompt closer follow-up and further assessment, including structured expectation management and pain-coping assessment, rather than any specific intervention. Poor preoperative mental health should not be viewed as a reason to discourage surgery, but rather as a clinical signal for a more tailored and multidisciplinary perioperative pathway. At present, there remains limited high-quality evidence supporting any specific prehabilitation strategy that effectively improves outcomes in these at-risk patients [42].

Among studies focusing on UKA, few have evaluated both patient satisfaction and MCID attainment [12, 14, 34]. Lee et al. found that poorer preoperative and postoperative MCS were associated with dissatisfaction at two years after UKA [43]. Our findings extend this observation and identify where the difference between mental health groups actually lies: not in knee-specific functional gain or MCID attainment, but in how patients perceived that recovery, with preoperative MCS independently predicting six-month satisfaction and expectation fulfillment. A possible explanation is that early dissatisfaction reflects a mismatch between preoperative expectations and the pace of postoperative recovery, particularly in the presence of persistent subacute pain and psychological distress [44, 45]. Patients with greater psychological distress may also be more susceptible to interpreting the precision and rapid-recovery narrative surrounding contemporary arthroplasty as an assurance of an immediate, pain-free result, leading to disappointment when recovery still requires time and rehabilitation; this remains a hypothesis that the present data cannot test.

These findings should be interpreted in light of several limitations. This was a retrospective, single-center study conducted in a predominantly Singaporean population, limiting the generalizability of the results. In addition, only about 50% of the 241 eligible UKAs met the complete-case inclusion criteria, introducing the potential for selection bias. However, sensitivity analyses between included (*n* = 121) and excluded (*n* = 120) patient cohorts showed similar demographic characteristics and preoperative PROMs, reducing the possibility of selection bias (Supplementary Table S4). Outcomes in the present study were assessed only up to two years postoperatively, and it remains unclear whether these findings will persist or change over longer follow-up. Therefore, larger prospective studies with longer-term follow-up are needed to validate these findings. Our study also did not account for preoperative radiographic osteoarthritis severity and comorbidities that could have confounded the present findings [46]. All patients were treated with robotic assistance and no conventional UKA comparator was available, so the present data describe the influence of preoperative mental health within a robotic-assisted cohort and cannot be used to evaluate the effect of the robotic platform itself. Numerous PROMs were also compared across three time points and within subgroups without formal adjustment for multiple comparisons, and the logistic regression models included few outcome events relative to the number of covariates; isolated significant findings and the regression estimates should therefore be interpreted cautiously. Finally, the trajectory subgroup analyses were exploratory, and comparisons of two-year MCS between trajectory subgroups are definitional rather than predictive.

Furthermore, ceiling effects in patients with high baseline MCS may have limited the detectable magnitude of postoperative mental health improvement. Although the SF-36 is a well-validated instrument that captures psychological symptoms overlapping with anxiety and depression, it was not designed to diagnose specific psychiatric disorders, and no patients in this cohort carried a formal diagnosis of a mental health disorder during the study period. Questionnaires specifically developed to assess anxiety and depression, such as the Beck Depression Inventory and Hospital Anxiety and Depression Scale, are not routinely collected in orthopaedic clinics, whereas the SF-36 is one of the most commonly used generic measures of health-related quality of life and is routinely collected in many orthopaedic practices. Accordingly, MCS is interpreted as a measure of general mental health and psychological distress [24–26], and not as a proxy for a diagnosis of depression, a construct that has itself been defined inconsistently in the literature [47].

## Conclusion

Within this robotic-assisted UKA cohort, poorer preoperative mental health was associated with inferior PROMs and lower satisfaction in the early postoperative period, yet these patients still achieved comparable gains in knee-specific function by two years, and no difference in satisfaction was detectable at two years. Poor preoperative mental health should not preclude appropriately selected patients from undergoing UKA. Assessment of mental health at six months may help identify patients who warrant closer monitoring; the associated MCS threshold was derived internally within a small subgroup and requires prospective external validation before it can be used to guide clinical decision-making.

## Supplementary Information

Supplementary Material 1.

## Data Availability

The data supporting the findings of this study are not publicly available due to privacy or ethical restrictions but are available from the corresponding author on reasonable request.
