# Supplementary material for: Preoperative mental health predicts early satisfaction but not two-year knee-specific functional improvement after unicompartmental knee arthroplasty: an analysis of a robotic-assisted cohort
Source: Arthroplasty. 2026 Nov 11;8:89. doi: 10.1186/s42836-026-00448-w (PMC13753461; doi:10.1186/s42836-026-00448-w)
Supplement: Supplementary file 1 [file 42836_2026_448_MOESM1_ESM.docx]

**Table S1.** Comparison of clinical outcomes between groups stratified by preoperative SF-36 MCS - expanded cohort (*n* = 241)

| **Outcome** | **MCS <50 (*n* = 93)** | **MCS ≥50 (*n* = 148)** | **Unadjusted**  ***p*-value** | **ANCOVA-adjusted** | | | | |
| --- | --- | --- | --- | --- | --- | --- | --- | --- |
|  |  |  |  | **MCS <50** | | **MCS ≥50** | | ***p*-value** |
| **OKS** | | | | | | | | |
| Preoperative | 35.94 ± 8.04 | 31.80 ± 8.01 | **<0.001** | - | | - | | - |
| 6 months | 20.42 ± 6.28 | 18.97 ± 5.74 | **0.037** | 20.13 ± 0.64 | | 19.15 ± 0.49 | | 0.227 |
| 2 years | 19.89 ± 6.26 | 17.17 ± 4.41 | **0.003** | 19.52 ± 0.67 | | 17.42 ± 0.55 | | **0.019** |
| Δ Preoperative to 6 months | −15.37 ± 9.80 | −12.92 ± 8.84 | 0.061 | - | | - | | - |
| Δ Preoperative to 2 years | −16.40 ± 8.88 | −14.27 ± 8.58 | 0.139 | - | | - | | - |
| Attained MCID at 6 months | 46 (93.9) | 55 (83.3) | 0.148 | - | | - | | - |
| Attained MCID at 2 years | 44 (89.8) | 57 (86.4) | 0.789 | - | | - | | - |
| **KSKS** | | | | | | | | |
| Preoperative | 39.04 ± 14.55 | 43.64 ± 17.96 | 0.075 | - | | - | | - |
| 6 months | 84.21 ± 18.75 | 87.71 ± 13.71 | 0.299 | 84.62 ± 1.80 | | 87.55 ± 1.39 | | 0.201 |
| 2 years | 81.90 ± 21.86 | 88.85 ± 13.76 | 0.148 | 81.58 ± 2.35 | | 88.72 ± 1.92 | | **0.021** |
| Δ Preoperative to 6 months | 45.84 ± 23.50 | 43.63 ± 20.99 | 0.214 | - | | - | | - |
| Δ Preoperative to 2 years | 42.98 ± 24.45 | 45.18 ± 23.13 | 0.661 | - | | - | | - |
| Attained MCID at 6 months | 46 (93.9) | 63 (95.5) | 0.699 | - | | - | | - |
| Attained MCID at 2 years | 41 (83.7) | 61 (92.4) | 0.243 | - | | - | | - |
| **KSFS** | | | | | | | | |
| Preoperative | 54.19 ± 18.58 | 60.98 ± 18.81 | **0.002** | - | | - | | - |
| 6 months | 73.82 ± 17.82 | 79.83 ± 14.65 | **0.009** | 74.53 ± 1.67 | | 79.39 ± 1.30 | | **0.023** |
| 2 years | 77.26 ± 16.66 | 82.17 ± 16.32 | 0.061 | 78.44 ± 2.00 | | 81.36 ± 1.65 | | 0.266 |
| Δ Preoperative to 6 months | 19.38 ± 22.66 | 19.03 ± 21.26 | 0.675 | - | | - | | - |
| Δ Preoperative to 2 years | 23.47 ± 23.41 | 21.17 ± 19.34 | 0.604 | - | - | | - | |
| Attained MCID at 6 months | 25 (51.0) | 28 (42.4) | 0.468 | - | - | | - | |
| Attained MCID at 2 years | 31 (63.3) | 52 (78.8) | 0.104 | - | - | | - | |
| **SF-36 PCS** | | | | | | | | |
| Preoperative | 34.63 ± 9.66 | 34.22 ± 8.88 | 0.921 | - | - | | - | |
| 6 months | 47.37 ± 10.11 | 49.59 ± 7.11 | 0.449 | 47.29 ± 0.87 | 49.64 ± 0.68 | | **0.034** | |
| 2 years | 47.75 ± 8.82 | 51.30 ± 7.80 | **0.002** | 47.74 ± 1.04 | 51.31 ± 0.87 | | **0.010** | |
| Δ Preoperative to 6 months | 12.52 ± 11.82 | 15.42 ± 10.55 | **0.045** | - | - | | - | |
| Δ Preoperative to 2 years | 12.76 ± 11.58 | 16.59 ± 11.72 | **0.032** | - | - | | - | |
| Attained MCID at 6 months | 35 (71.4) | 51 (77.3) | 0.62 | - | - | | - | |
| Attained MCID at 2 years | 34 (69.4) | 56 (84.8) | 0.079 | - | - | | - | |
| **Patient satisfaction** | | | | | | | | |
| Satisfied at 6 months | 40 (81.6) | 64 (97.0) | **0.009** | - | - | | - | |
| Satisfied at 2 years | 45 (91.8) | 65 (98.5) | 0.162 | - | - | | - | |
| **Patient expectation** | | | | | | | | |
| Expectation fulfilled at 6 months | 34 (69.4) | 59 (89.4) | **0.014** | - | - | | - | |
| Expectation fulfilled at 2 years | 42 (85.7) | 62 (93.9) | 0.2 | - | - | | - | |

Data presented as mean ± standard deviation or n (%). Bold *p-*values indicate statistical significance. Δ represents the change from preoperative to 6 months or 2 years. OKS - lower scores indicate better outcomes. ANCOVA-adjusted analyses were presented with adjusted means and standard errors (SE) estimated at the grand mean of the corresponding baseline covariate, age, sex, and BMI.

ANCOVA, analysis of covariance; BMI, body mass index; KSKS, Knee Society Knee Score; KSFS, Knee Society Function Score; OKS, Oxford Knee Score; MCID, minimal clinically important difference; MCS, mental component summary; PCS, physical component summary; SE, standard error; SF-36, 36-item Short-Form health survey.

**Table S2.** Multivariable logistic regression for achieving patient satisfaction - expanded cohort

| **Variable** | **OR** | **95% CI** | | **95% CI** | ***p*-value** |
| --- | --- | --- | --- | --- | --- |
|  |  | **Lower** | **Upper** |  |  |
| **Satisfied at 6 months (*n* = 228, Yes = 205, No = 23)** | | | | | |
| Preoperative SF-36 MCS | 1.048 | 1.011 | 1.086 | 1.011–1.086 | **0.011** |
| Age | 1.061 | 0.998 | 1.127 | 0.998–1.127 | 0.058 |
| Sex (Women) | 1.182 | 0.427 | 3.268 | 0.427–3.268 | 0.748 |
| **Sex (Men)** |  |  |  |  |  |
| BMI | 1.098 | 0.984 | 1.226 | 0.984–1.226 | 0.094 |
| **Satisfied at 2 years (*n* = 151, Yes = 146, No = 5)** | | | | | |
| Preoperative SF-36 MCS | 1.056 | 0.990 | 1.127 | 0.990–1.127 | 0.099 |
| Age | 1.079 | 0.951 | 1.223 | 0.951–1.223 | 0.239 |
| Sex (Women) | 0.591 | 0.088 | 3.978 | 0.088–3.978 | 0.589 |
| **Sex (Men)** |  |  |  |  |  |
| BMI | 1.086 | 0.871 | 1.356 | 0.871–1.356 | 0.463 |

Bold *p-*values indicate statistical significance. Bold variable labels denote the reference category.

BMI, body mass index; CI, confidence interval; MCID, minimal clinically important difference; MCS, mental component summary; OR, odds ratio; SF-36, 36-item Short-Form health survey.

**Table S3.** Multivariable logistic regression for achieving expectation fulfillment - expanded cohort

| **Variable** | **OR** | **95% CI** | | **95% CI** | ***p*-value** |
| --- | --- | --- | --- | --- | --- |
|  |  | **Lower** | **Upper** |  |  |
| **Expectation fulfilled at 6 months (*n* = 230, Yes = 195, No = 35)** | | | | | |
| Preoperative SF-36 MCS | 1.037 | 1.006 | 1.070 | 1.006–1.070 | **0.020** |
| Age | 1.047 | 0.996 | 1.101 | 0.996–1.101 | 0.072 |
| Sex (Women) | 1.281 | 0.553 | 2.965 | 0.553–2.965 | 0.564 |
| **Sex (Men)** |  |  |  |  |  |
| BMI | 1.058 | 0.966 | 1.158 | 0.966–1.158 | 0.225 |
| **Expectation fulfilled at 2 years (*n* = 151, Yes = 139, No = 12)** | | | | | |
| Preoperative SF-36 MCS | 1.040 | 0.990 | 1.092 | 0.990–1.092 | 0.102 |
| Age | 0.985 | 0.909 | 1.067 | 0.909–1.067 | 0.711 |
| Sex (Women) | 0.592 | 0.156 | 2.244 | 0.156–2.244 | 0.44 |
| **Sex (Men)** |  |  |  |  |  |
| BMI | 1.201 | 1.014 | 1.423 | 1.014–1.423 | **0.034** |

Bold *p-*values indicate statistical significance. Bold variable labels denote the reference category.

BMI, body mass index; CI, confidence interval; MCID, minimal clinically important difference; MCS, mental component summary; OR, odds ratio; SF-36, 36-item Short-Form health survey.

**Table S4.** Comparison of baseline demographics and preoperative outcomes between included and excluded patients

| **Variable** | **Included**  **(*n* = 121)** | **Excluded**  **(*n* = 120)** | ***p*-value** |
| --- | --- | --- | --- |
| Age, years | 63.9 ± 8.6 | 64.6 ± 7.6 | 0.432 |
| BMI, kg/m² | 26.9 ± 4.6 | 27.1 ± 3.8 | 0.551 |
| **Sex** | | | |
| Women | 90 (74.4) | 76 (63.3) |  |
| Men | 31 (25.6) | 44 (36.7) | 0.064 |
| **Race** | | | |
| Chinese | 97 (80.2) | 94 (78.3) |  |
| Malay | 16 (13.2) | 14 (11.7) |  |
| Indian | 6 (5.0) | 9 (7.5) |  |
| Others | 2 (1.7) | 3 (2.5) | 0.778 |
| **Preoperative outcomes** | | | |
| OKS | 33.0 ± 8.1 | 33.7 ± 8.6 | 0.441 |
| KSFS | 59.2 ± 18.8 | 58.7 ± 19.8 | 0.936 |
| KSKS | 41.7 ± 15.8 | 43.0 ± 17.1 | 0.607 |
| SF-36 PCS | 35.2 ± 9.3 | 33.9 ± 9.5 | 0.246 |
| SF-36 MCS | 51.0 ± 11.1 | 51.9 ± 11.5 | 0.342 |

Data presented as mean ± standard deviation or n (%).

BMI, body mass index; KSKS, Knee Society Knee Score; KSFS, Knee Society Function Score; OKS, Oxford Knee Score; MCS, mental component summary; PCS, physical component summary; SF-36, 36-item Short-Form health survey
